# The Energy Transition between Desideratum and Challenge: Are Cogeneration and Trigeneration the Best Solution?

**DOI:** 10.3390/ijerph19053039

**Published:** 2022-03-04

**Authors:** Adrian Neacșa, Mirela Panait, Jianu Daniel Mureșan, Marian Catalin Voica, Otilia Manta

**Affiliations:** 1 Department of Mechanical Engineering, Petroleum-Gas University of Ploiesti, 100680 Ploiești, Romania; adineacsa@gmail.com; 2 Department of Cybernetics, Economic Informatics, Finance and Accounting, Petroleum-Gas University of Ploiesti, 100680 Ploiești, Romania; 3 Institute of National Economy, 050771 Bucharest, Romania; 4 Department of Business Administration, Petroleum-Gas University of Ploiesti, 100680 Ploiești, Romania; jianu_muresan@yahoo.com; 5 Financial and Monetary Research Center “Victor Slavescu”, Romanian Academy, 050711 Bucharest, Romania; otilia.manta@rgic.ro; 6 Department of Research, Romanian-American University, 012101 Bucharest, Romania

**Keywords:** energy transition, cogeneration, trigeneration, energy efficiency, sustainable development

## Abstract

Climate change has brought to the attention of politicians, researchers, and other stakeholders the need to protect the environment. The concerns at the international level are more and more intense, and the solutions found are multiple. One of the directions to follow is a new energy transition, which involves the use of renewable energy, but also techniques of cogeneration and trigeneration. This study presents the main research on increasing energy efficiency in the use of a primary energy source and the impact on the environment. Compared to the classical methods of obtaining heat and electricity from burning fossil fuels through separate technologies, the study brings to the fore two methods (cogeneration and trigeneration) that have much higher yields by obtaining two or even three forms of energy from the use of a single source of combustion. The impact on the environment is also significantly reduced by eliminating additional sources of pollution (reducing greenhouse gas emissions). Taking into account the evolutions of the energy market during this period, this article aims to analyze, from the point of view of the two most important influencing factors, the economic efficiency of processes and the reduction of greenhouse gas emissions by overlapping their effect, in case of the use of modern technologies (cogeneration and trigeneration), for the combined production of various forms of energy.

## 1. Introduction

Energy production and consumption are being intensely debated informally, but also academically, considering the relationship between energy, economic growth, and sustainable development [1,2,3,4,5,6,7,8]. The increase in environmental concerns has led to the intensification of technical innovations to reduce energy consumption (creating high-efficiency equipment) and to produce green energy, which are accompanied by financial and social innovations, but also by changing consumer attitudes and behavior towards various categories of renewable energy [9,10,11,12]. Reducing energy consumption is a goal pursued by companies, public authorities, and other stakeholders, but researchers have drawn attention to the risks posed by lower energy prices and increased energy efficiency that causes the Jevons paradox [13,14,15]. For this reason, public policies to increase energy efficiency must be configured to take into account the multiple forms of the rebound paradox; there are three types of paradox: direct, indirect, and economy-wide.

In addition, the fast paces of industrialization, but also of urbanization, specific to emerging countries generate an increase in electricity consumption with direct consequences on environmental pollution [7,9,16]. For this reason, more and more studies are focusing on the Environmental Kuznets Curve and the processing of statistical data for different groups of countries, demonstrating the existence of an inverted U-shaped relationship between environmental pollution (usually measured as carbon dioxide emissions) and economic development [17,18,19,20]. Economic development generates, in the first phase, an increase in energy intensity. Given the negative externalities generated by the increase in energy consumption for the creation of goods and services, public authorities are becoming increasingly concerned about the decline of energy intensity. Studies conducted worldwide have shown substantial progress made by EU countries in this area given the specifically promoted economic policies [21,22,23,24,25,26,27]. The decrease in energy intensity is a desideratum for all countries; the measures that can be adopted are multiple and complex, the ideal being the increase of convergence in this field. Changing the structure of the economy can be a solution by transitioning from more energy-intensive industries to less energy-intensive sectors. Another solution is the emergence of energy-saving technologies. Unfortunately, energy savings obtain as a result of national progress by increasing the energy efficiency of equipment can be substantially reduced by behavioral factors, demographic changes related to population aging or other factors, such as global warming, which generates an increase in energy consumption during the summer.

**Figure 1:**
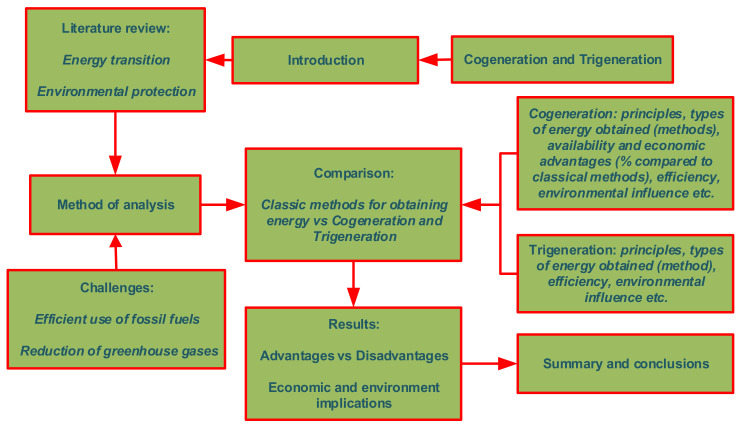
General chart of the present study. Source: the authors based on selected scientific studies.

The aim of this paper is to analyze the economic solutions (availability and efficiency) and environmental protection (reduction of greenhouse gas emissions) offered by cogeneration and trigeneration in the complex process of energy transition, in which all states of the world have entered given the challenge generated by global warming. Protecting the environment is a goal that can be achieved in multiple ways, and the energy transition can be one solution, but the economic, social, technical, and even political challenges specific to this process are multiple and interdependent. This study presents the main research on increasing energy efficiency in the use of a primary energy source and the impact on the environment. Compared to the classical methods of obtaining heat and electricity from burning fossil fuels through separate technologies, the study brings to the fore two methods (cogeneration and trigeneration) that have much higher yields by obtaining two or even three forms of energy from the use of a single source of combustion. This article is organized into four sections (see Figure 1): Introduction, Literature review, Method of Analysis, and Conclusions. After the Introduction, the authors present, in the Literature review section, the main results of the studies regarding the energy transition processes in different countries and regions and specific solutions for a smooth energy transition. In the main section, the authors present a comparison of the cogeneration and trigeneration processes, and specific indicators proposed in order to reveal the low impact of these processes on the environment. The article ends with a section of conclusions, where some proposals for economic policy measures, research limits, but also future research directions, are presented.

## 2. Literature Review

Five years after the initiation of the Paris Climate Agreement, the year 2020 became a milestone through The European Green Deal. One of the fundamental theses of the two treaties (with a major strategic target for 2050, but with explicitly formulated objectives for the 2030 horizon) is the energy transition. The energy transition involves the replacement of energy production and consumption models based on fossil fuels (oil, natural gas, and coal) with systems that use renewable resources [28]. This complex process is generated by numerous factors, such as climate change and the increase in the price of fossil fuels because of the reduction of deposits that can be exploited under the current conditions of economic efficiency. The current energy transition is a profound transformation that involves a complex and lengthy process that raises several issues related to the feasibility and viability of the transition, ways to measure the stage and potential of the transition, and the threats posed by the pandemic crisis. The complexity of this phenomenon has generated widespread concern from many researchers, who even developed a theory of energy transition given the characteristics of the first energy transitions (from wood and waterpower to coal in the 19th Century, or from coal to oil in the 20th Century), which generated numerous economic, social and environmental consequences, such as industrialization, urbanization, climate change, and the emergence and development of the consumer society [29].

Researchers, such as Geels, 2002, 2005; Verbong and Geels, 2007; Geels and Schot, 2007; and Verbong et al., 2008, using concepts from related fields, such as evolutionary economics, sociology and the history of technology, and political economics, proposed a multi-level perspective (MLP) for energy transition, which consisted of three inter-linked processes on micro, medium, and macro levels: (1) at the micro level, new technologies or energy types, such as biofuels and nuclear power, are used (niche-innovations); (2) at the medium level, a network of different categories of stakeholders is set up, which acts according to specific formal, normative, and cognitive rules; (3) at the macro level, the socio-technical landscape is metamorphosed through behavioral changes among consumers and companies, and public authorities that create the legal and institutional framework for the promotion and use of new energy sources.

Therefore, the energy transition is a long process that involves behavioral changes for consumers, companies, and public authorities, an intense process of technical innovation, the development of mechanisms and financial products specific to the energy market, and the adoption of legal regulations to provide directions for action for different categories of stakeholders [30,31,32].

### 2.1. The Problem of Feasibility and Viability

Debates about the feasibility of a system that uses 100% renewable sources to produce electricity (100% renewable electricity system, 100% RE system) are far from over. While some authors unreservedly support this model [3,33,34,35], others try to demonstrate that feasible and sustainable 100% RE systems are just myths [36].

One of the great challenges of the energy transition is managing it so that it does not generate social inequalities. The transition must not create inequity and disadvantage for the poorest in favor of the richest, as has happened in previous industrial transitions. Subsidy policies of a sector using fiscal leverage, injecting public funds, or controlling prices only redistribute wealth from one category of society to another. On the other hand, giving up subsidizing industrial sectors that use fossil fuels can lead to higher prices and lower consumer purchasing power.

In addition, one of the acute problems is access to energy. Globally, more than 800 million people (mostly in sub-Saharan Africa) have no access to electricity at all. Energy poverty is a complex phenomenon that affects citizens in both developed and developing countries, both in warm and temperate areas. Energy poverty is a concern not only for researchers, but also for public authorities given the multiple social, medical, and environmental consequences it has [37,38]. Although the current energy transition strategy is mainly based on concerns about environmental sustainability and global warming, a strategic component with the same weight should take into account access and energy security, and be able to ensure economic prosperity and human development. In addition, the expansion of the use of renewable sources will generate the emergence of trades and jobs. The estimates made by the International Renewable Energy Agency (IRENA) regarding the jobs associated with renewable energy are around 16.7 million by 2030 [39]. In addition, the new energy projects also contribute to the development of local communities through the training effects that any investment generates. Unfortunately, the externalities are accompanied by negative effects, such as the resistance of the inhabitants and their emotional reactions to certain aspects related to such investment, namely the pollution potential of the area, changing the landscape of the locality [40].

Therefore, the energy transition is a phenomenon with multiple consequences.

- “Hard to abate” sectors. Globally, about 73% of greenhouse gas emissions come from energy generation or consumption, most of which represent CO_2_ emissions. Of these, 53% result from energy production and road transport. The remaining 47% come from the activity of economic sectors called “hard to abate”: the metallurgical and steel industry (approx. 6.5% of global CO_2_ emissions), chemical industry (approx. 3%), aviation sector (approx. 2.7%), and naval transport (approx. 2.6%) [41]. For the near future, there will no be viable solutions for these sectors, except, perhaps, green hydrogen.
- “Intermediate and alternative solutions”. Power-to-X is a term that covers a wide range of technological processes by which electricity is converted into heat, hydrogen, and synthetic fuels. Green hydrogen is produced using renewable energy (wind and solar), which, by electrolysis, will separate water molecules into their constituent parts. In addition, green hydrogen can be stored and saved, and a process of adding carbon dioxide can produce the synthetic fuels needed to “decarbonize” the “hard to abate” sectors and supply chains. For now, the main obstacle in generalizing Power-to-X solutions is the cost.

Energy efficiency is the fundamental solution in the process of building sustainable and secure energy systems. Currently, only 33% of the primary energy used is converted into useful energy [41]. The remaining 67% represent losses due to the inefficiency of generation and transport facilities, heavy industries, and the energy inefficiency of buildings. In this sense, cogeneration and trigeneration can be viable solutions, as they result from the use of highly efficient energy installations, and different forms of energy are produced in a single installation by burning the same amount of fuel; in this way, significant reductions of pollutant emissions can be registered. However, when increasing efficiency or using alternative resources is not possible, the solution is to capture carbon by mechanical means, as in the case of the production of “blue hydrogen”, when the resulting carbon dioxide is compressed, transported from where it was produced, and injected into underground geological formations.

### 2.2. Threats Posed by the Pandemic Crisis

In addition to the long-term uncertainties generated, the shock caused by the pandemic crisis exerted immediate effects on the energy transition in 2020:

- Decreasing global energy demand by a third. As many businesses temporarily or permanently disrupted their operations for security reasons, supply chains were blocked. A major contribution was made by China, a major producer of renewable energy, where restrictions had a cascading effect on blocking the global value chain;
- Unprecedented volatility in oil and gas prices with various geopolitical implications;
- Delay or cancellation of investment projects. After 20 years of constant growth of investments in renewable energy-generating projects, the year 2020 marked a stagnation and even a decrease in certain segments. The worst-affected was Europe, where restrictive lockdown conditions pushed investors away and delayed auctions. Solar and wind energy, which account for the largest share of renewable capacity in Europe, fell by 12% and 21% respectively [42];
- The labor market suffers, on the one hand, due to uncertainties related to job security for millions of employees in the energy sectors, and, on the other hand, due to the labor shortage caused by traffic restrictions and even the closure of borders for foreign workers/migrants.

### 2.3. The Problem of Assessing the Stage of Transition

As the concept of energy transition is inter- or even cross-disciplinary, measuring the stage of transition requires a complex approach from a multitude of perspectives. A number of global, regional, or national organizations record specific statistical indicators regarding the efficiency of energy systems, the demand and supply of energy from renewable sources, the level of investments, the level of greenhouse gas emissions, the level of energy prices, etc. There have also been multiple attempts to generate aggregate indicators to provide an overview of the phenomenon. Here are the most important achievements in this regard (see Table 1).

Of the stated options, the most relevant (but obviously perfectible) is the Energy Transition Index (ETI). This index provides a broader conceptual framework, aggregating a series of sub-indices under the tutelage of two major dimensions: System Performance and Transition readiness (see Table 2). The choice of dimensions, variables, sub-indices, and their weights in the final score resulted from the feedback generated by the consultation of stakeholders from different countries.

ETI covers 115 countries, representing about 90% of the world’s population and over 90% of nominal GDP. The 40 indicators taken into account will generate a score for each country from 0 to 100.

**Figure 2:**
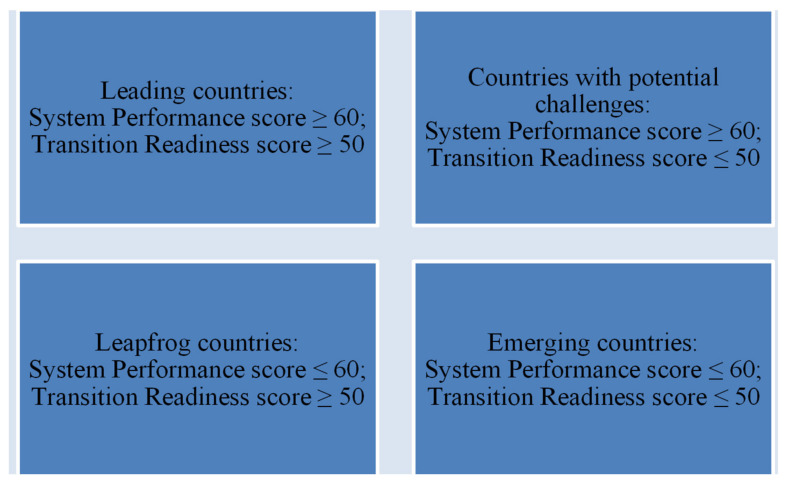
Categories of countries with two dimensions of the TSI. Source: authors based on Singh et al., (2019) [43].

The score obtained by a country is influenced by a number of factors, such as the level of economic and social development, industrial structure, population and consumption habits, geography and climate, available natural resources, the state of the global energy market (stability/volatility), the evolution of the legislative framework of trade partners in the field of environmental protection, etc. As can be seen in Table 3, no country reached the maximum score of 100. The average score calculated for the 115 countries included in the ranking was 55.1. Singh et al., (2019) identified four categories of countries (see Figure 2), depending on the values of the scores for the two dimensions of the TSI (System Performance and Transition Readiness) [43].

Even if the objectives of the Paris Agreement are achieved, through the major contribution of the “Leading” states, the energy transition will not be complete. The ETI scores indicate that, at present, 56 countries (Countries with potential challenges and Emerging countries) are not prepared for the energy transition. As it results from Table 3, 48 countries are part of the “Leading countries” category, the vast majority of them (30) being European states. The average ETI score for the top 10 countries has remained relatively constant over the last five years. The “Leapfrog countries” category includes 11 states, including China and the United Arab Emirates. It is considered that these countries are not currently performing, but have both the availability and the potential to achieve the energy transition. Seventeen states are considered “Countries with potential challenges”. Countries such as the Russian Federation, the Philippines, Brazil, and Argentina have efficient energy systems, sufficient resources available, but the legislative framework, governance, and institutional framework do not support investment, innovative business, and the involvement of human capital in the energy transition. Thirty-nine countries are classified as “Emerging countries”, with low scores both for the current performance of their energy systems and for their availability to the energy transition. Most states in this category are located in North and Sub-Saharan Africa, but also in the Middle East and Latin America. In addition, the category includes Turkey, several European countries, such as Poland, Bulgaria, or countries of former Yugoslavia (such as Serbia), but also a number of heavily populated Asian countries (India and Bangladesh).

Fossil fuel-based systems will work and coexist with renewable energy-based systems for at least two generations from now. Therefore, no matter how important the concern to invest and implement the energy transition, until its completion, a major strategic component must consider the efficiency of models that use fossil fuels in order to reduce costs, but also carbon emissions. In this sense, cogeneration and trigeneration can be part of the solution now, waiting for the next transition.

## 3. Method of Analysis

This chapter describes the two technological processes for simultaneously obtaining energy forms in terms of process parameters. The analysis method takes into account the two most important influencing factors, the economic efficiency of the processes and the reduction of greenhouse gas emissions. By overlapping their effects, it will be possible to consider the usefulness of the two modern technologies (cogeneration and trigeneration) to obtain different forms of energy.

The rational use of energy has fluctuated as a result of the oil crises of the 1980s. From this point of view, energy production has become a major part of humanity. This current case study presents the main research on increasing energy efficiency in the use of a primary energy source and the impact on the environment. Compared to the classical methods of obtaining heat and electricity from burning fossil fuels through separate technologies, the current case study brings to the fore two methods (cogeneration and trigeneration) that have much higher yields by obtaining two or even three forms of energy from the use of a single source of combustion. The impact on the environment is also significantly reduced by eliminating additional sources of pollution (reducing greenhouse gas emissions).

The primary energy sources are of three types:

- Based on fossil fuels: coal, oil, gas, and wood;
- Nuclear energy, which does not produce greenhouse gases when producing usable energies (electric or thermal), but contributes to pollution through the thermal energy released in the cooling environments. In addition, nuclear waste creates storage problems, having a negative effect on the environment;
- “Clean” green energies, non-polluting, and inexhaustible: solar energy, energy produced by wind, and energy produced by the force of water.

Currently, worldwide, the main energy resource (about 70%) is fossil fuels (the first of the three categories mentioned above). Increasing energy consumption in conjunction with the depletion of fossil fuel resources and the need to reduce environmental pollution due to greenhouse gas emissions or radioactive waste involves the increasing use of renewable energy [45].

In addition, there are various possibilities for the revaluation of energy used in other technological, civil, or environmental processes: the energy of fluids resulting from industrial (flue gases, condensation, and hot water) or civil processes (domestic hot water); urban and rural or forest waste that can be burned directly or can produce biogas; ground energy, groundwater, or geothermal energy; and the energy of the outside or discharged air from an enclosure.

All of these forms of production and the more economical and efficient use of energy forms are priority research directions of society. At the same time, it is very important to reduce energy consumption at both levels, industrial and domestic. The increase in the price of fossil fuels and, consequently, of thermal and electrical energy, as well as the introduction of restrictions on greenhouse gas emissions, lead to the identification of methods to improve the energy efficiency of installations, contributing to low energy consumption [45]. The EU directives on energy end-use efficiency are binding on all Member States of the European Union and provide for the commitment of EU Member States to reduce final energy consumption.

In this context, a comparative case study was conducted on the use of two methods of obtaining, from burning hydrocarbons, forms of energy using cogeneration (thermal and electrical energy) and trigeneration (thermal, electrical, and cooling energy). Absorption refrigeration installations are studied because they are receiving increased interest among researchers [46,47] due to the fact that they are driven by thermal energy that may come from a renewable source waste: hot water heated by solar energy, geothermal water, hot water resulting from a thermal power plant, flue gases resulting from industrial processes, or heat engines from cogeneration plants. In this way, energy savings can be achieved and CO_2_ emissions into the atmosphere can be reduced. Thus, absorption plants are an energy-efficient and less-polluting alternative to convert cheap heat from cogeneration plants into cooling or heat.

Absorption systems are important in the field of cooling and air conditioning production and in the field of heat production when refrigeration systems are transformed into heat pumps. Compared to the use of cogeneration plants, from which only two forms of energy, heat and electricity, can be obtained, applications for absorption plants can range from those for small-capacity domestic air conditioning to industrial ones with the biggest capacity. Ammonia-water absorption plants are one of the oldest technologies for obtaining cooling, being used first by Ferdinand Carré in 1859. In the middle of the last century, through the greater development of the simplest systems based on the use of electricity (refrigeration systems that use mechanical compressors), there has been a decrease in applications based on absorption installations. In the last three decades, there has been a growing interest in research into absorption systems with the awareness of energy saving and environmental protection issues. From a research perspective, they are an area of continuous development, linked to the current requirements of rational and efficient use of energy and reducing polluting effects on the environment. The main concerns of engineers are:

- The use of systems based on operating cycles as efficient as possible;
- Improving equipment performance;
- The use of working fluids as adapted as possible to the use of renewable or recovered energy sources, being less polluting and more efficient;
- Extending the range of thermal powers, especially to small ones, for use in commercial or domestic applications;
- Increasing the possibility of adjustment, automatic control, and reliability.

All of these concerns are not limited only to the improvement of technologies for the construction of these installations, in order to increase work efficiencies and reduce pollution, but are extended and correlated with the economic side; therefore a technical and economic optimum of these is always sought in engineering solutions.

Considering the above, the two engineering solutions with the advantages of using the same primary energy source to obtain two or three energy components will be presented below. When technology combines electricity, heating, and cooling production from one source, carbon emissions are reduced significantly.

For all organizations seeking to reduce operating costs and improve power efficiency, as well as a reduction in carbon emissions, a number of new likely technologies are presented in Table 4.

It is also very important to pay attention to energy efficiency. The energy efficiency of global primary resources represents a percentage of about 40% of the total reduction potential of carbon dioxide and other greenhouse gases, which can be achieved with the involvement of relatively low costs. These types of technological systems can be used in combined variants and can be among the best options, achieving an optimum that is difficult to match between the low cost level and the efficiency of the transformation ratio.

Both technologies are used in many facilities, such as destination buildings:

- Commercial (commercial office buildings, SPAs, hotels and restaurants, orphanages, and nursing homes);
- Residential (living spaces, apartments, and neighborhoods);
- Local authorities (centralized energy systems, and utility installations);
- Institutions (educational spaces, hospitals, barracks, and prisons);
- Companies (factories for products/services in all industries) etc.

Technologies for obtaining thermal energy from renewable/sustainable sources are safe, efficient, environmentally friendly, and increasingly competitive in terms of reducing cost levels to reduce the use of conventional energy. The technologies are verified, mature, and low risk, offering significant and complex benefits (financial, environmental, and energy) to co-stakeholders. Heating and cooling processes using renewable/sustainable sources offer the following benefits [49]:

- Predictable and often fixed-price energy levels;
- Reduction of costs with the use of conventional energy sources (wood, coal, oil, and natural gas);
- Reducing emissions of carbon dioxide and other greenhouse gases without affecting performance or comfort;
- The use of sustainable/renewable resources to the detriment of conventional fuels;
- Energy independence through the development of internal energy sources;
- Stimulating local job creation with implications for domestic economic growth.

**Figure 3:**
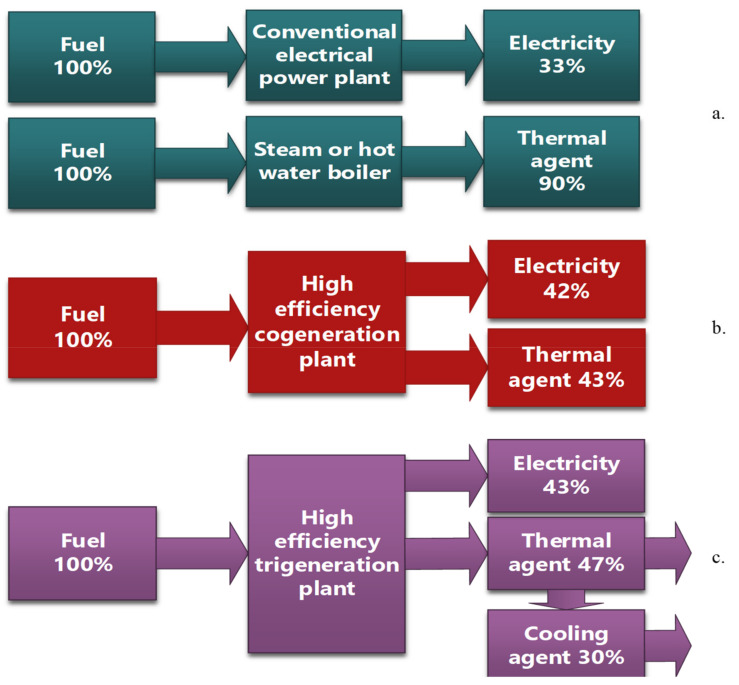
Energy efficiency for different energy production processes. (**a**) Process efficiency 61.50% ((33 + 90)/200 = 0.615); (**b**) process efficiency 85% ((42 + 43)/100 = 0.85); (**c**) process efficiency up to 90% ((43 + 47 (30% − cooling agent))/100 = 0.90).

**Figure 4:**
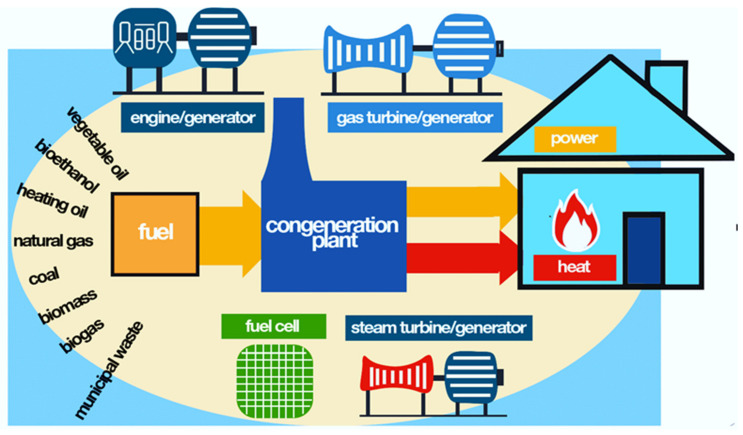
Cogeneration principle. Source: www.cogeneurope.eu (accessed on 20 October 2021).

We present the efficiency of the production processes of various forms of energy (see Figure 3 and Figure 4).

In industrial applications, different types of CHP technological systems can be identified that use different primary energy sources, which modify the way the residual heat is collected [49,50,51]. The types of CHP technology systems include:

- Steam Turbine/Boiler-these systems primarily produce energy using combustion sources, such as gas, coal, biomass, digester gas, and refinery off-gas [49,50,51];
- Combined Cycle-these systems primarily produce energy using conventional energy source, such as coal [49,50,51];
- Combustion Turbine-these plants primarily produce power using natural gas and propane [51];
- Fuel Cell-these plants primarily produce power using fossil sources, such as oil, jet fuel, distillate fuel oil, residual fuel oil, and kerosene [49,50,51];
- Micro turbine-these systems primarily produce energy using renewable sources like waste, waste heat, blast furnace gas, municipal solid waste (MSW), petroleum coke, black liquor, and process gas [49,50,51];
- Biofuel Engine-these systems primarily produce energy using a reciprocating engine adapted to use renewable sources, such as biofuel, for combustion [49,50,51].

In the following, as can be seen in Table 5 and Table 6, a comparison was made between some of the cogeneration energy production systems with the best available and economically justifiable technology for the separate production of heat and electricity on the market presented above.

As can be seen in Table 5 and Table 6, the yields for obtaining the two forms of energy through the cogeneration method are very good. Additionally, by applying this method to the same amount of fuel consumed, the same noxious volume will result in the atmosphere as in the case of obtaining a single form of energy.

The method involving the trigeneration process (CCHP—Combined Thermal, Cooling, and Power) is an innovative technology to obtain combined and simultaneously three types of energy from the use of a single fuel source. Additionally, in addition to the production of thermal and electrical energy (as in the case of the cogeneration process), a percentage of cooling energy is also obtained. Trigeneration technology includes the classic components of a cogeneration plant with an additional absorption cooling circuit and its specific accessories (the part that generates the cooling energy). Absorption cooling systems are the oldest environmentally friendly technological variants of cooling [52].

The percent of residual heat recovered in cogeneration plants is, in fact, the principal source of energy used in the cooling process in the trigeneration plants. For the cooling process, it can be concluded that no additional source of electricity or use of additional fuel is required, unlike other cooling technologies (electric coolers or gas heat pumps). In this case, the only electricity intended for the cooling process is that used by the peripherals in the absorption cooling system (controllers, sensors, pumps, lights, etc.) [52].

The technological process of trigeneration can be considered an update to cogeneration plants both in terms of the technology used and its efficiency. In terms of the technology used, the process adds more functionality to the system, and in terms of efficiency, it is found that it allows potential savings of even greater amounts of electricity. The question is “Why is that?” In accordance with the principle of energy efficiency, it is necessary that no amount of energy is wasted, and everything that is generated must be completely consumed. Additionally, from the point of view of engineers and economists, there must always be a technical-economic optimum between the technology used and the quantities of electricity, heating. and cooling obtained. From the analysis of the aspects presented above, it can be highlighted that, if the thermal energy resulting from the processes in the cogeneration plants is used during the winter only for heating the built volumes and for domestic hot water throughout the year, the need for this type of energy in seasons with higher temperatures is substantially lower, so the generation of thermal energy should be reduced and electricity production should be increased. The process of transforming the heat resulting from the process into cooling energy used for air conditioning will allow the system to operate at full power throughout the year [52].

The trigeneration systems (CCHP) are primarily intended for energy consumers in the industrial and service sectors, who use large amounts of cooling energy in manufacturing processes or in the central air conditioning of large spaces [52].

Additionally, the trigeneration systems (CCHP) always generate three types of energy at the same time (thermal, electrical, and cooling), but, nevertheless, they may be designed to supply the consumer with all forms of energy or, where applicable, any of the three [52].

The logical order of energy production in cogeneration and trigeneration systems, when only two of them are required, always requires that electricity be paramount, while the difference is allocated to thermal energy in the case of cogeneration systems and cooling energy in the case of trigeneration systems. Consumer-specific energy needs impose the quantitative level as well as the types of energy required. The required amount of energy is also influenced by a significant number of technical requirements, which must be carefully considered before designing, building, and using such a system [52].

The increasing use of cogeneration (CHP) and trigeneration (CCHP) systems as important sources of energy generation required in manufacturing or service delivery processes could lead to a reduction in both pollutant emissions and substantial energy savings compared to those resulting from separate production (electricity, heat, and cooling). The major advantage in terms of saving on primary/conventional energy is also visible.

However, it is necessary to further investigate the potential for reducing hazardous greenhouse gas (GHG) emissions from both methods (cogeneration and trigeneration). A new method for estimating the impact of pollution on the environment is proposed in the literature, based on an indicator that highlights the reduction of CO_2_ emissions by using trigeneration (*TCO_2_ER*) and assesses the level of reduction of carbon dioxide and other emissions of GHG-type pollutants that result from the cogeneration and trigeneration processes, in comparison with their separate production (thermal energy, electricity, and cooling). This indicator is defined in accordance with the level of performance characteristics specific to the cogeneration and trigeneration systems, represented by systemic operation schemes (systemic models) in accordance with the characteristics of GHG emissions from processes using conventional sources [47,53].

**Figure 5:**
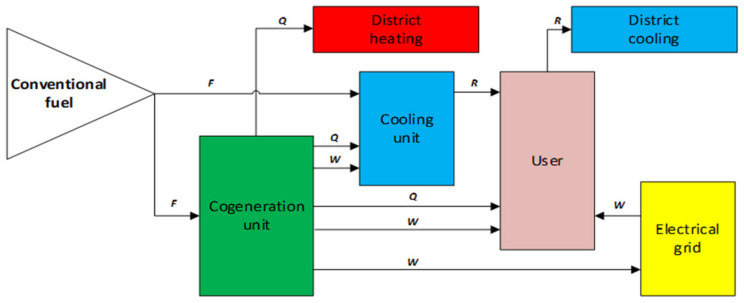
Trigeneration P&ID (trigeneration technological scheme and flows of the energy). *F*-fuel thermal content (kWht); *Q*-heat (kWht); *R*-cooling (kWht); *W*-electricity (kWhc).

In order to synthetically understand the trigeneration process, it will be presented schematically in the form of a system (using blocks as subsystems) how to obtain from a single combustion of a conventional fuel the three types of energy (see Figure 5).

Studies have shown that there are two indicators that best describe the operating characteristics and the level of pollutant emissions for both solutions for obtaining several types of energy from consuming a single fuel, i.e., cogeneration and trigeneration.

*TCO_2_ER* is an indicator relevant for the evaluation of the reduction of carbon dioxide emissions in trigeneration (and cogeneration) systems. *TCO_2_ER* is currently defined as:

(1) $${TCO}_{2}ER=\frac{{\left(m_{{CO}_{2}}\right)}_{SP}-{\left(m_{{CO}_{2}}\right)}_{z}}{{\left(m_{{CO}_{2}}\right)}_{SP}}$$

where:

${\left(m_{CO_{2}}\right)}_{z}$-the carbon dioxide mass resulting from the combustion of the fuel used in the trigeneration process;

${\left(m_{CO_{2}}\right)}_{SP}$-the carbon dioxide mass resulting from the production of the same amount of energy from trigeneration in relation to their separate production (*SP*).

*TCO_2_ER* can be expressed much more clearly using relevant emission terms and factors as in the relation:

(2) $${TCO}_{2}ER\;=1-\frac{{\left(\mu_{{CO}_{2}}^{F}\right)}_{z}\cdot F_{z}}{{\left(\mu_{{CO}_{2}}^{W}\right)}_{SP}\cdot W_{z}+{\left(\mu_{{CO}_{2}}^{Q}\right)}_{SP}\cdot Q_{z}+{\left(\mu_{{CO}_{2}}^{R}\right)}_{SP}\cdot R_{z}}$$

where ${\left(\mu_{CO_{2}}^{W}\right)}_{SP}$, ${\left(\mu_{CO_{2}}^{Q}\right)}_{SP}$, and ${\left(\mu_{CO_{2}}^{R}\right)}_{SP}$ are the emission factors in accordance with the energy production for the energy production systems (thermal, electrical, and, respectively, cooling).

These emission terms are conventionally established. The term ${\left(\mu_{CO_{2}}^{F}\right)}_{z}$ refers to the specific quantity of fuel introduced in the process of trigeneration. The cogeneration process (CHP) can be analyzed as a sub-case under limit conditions, corresponding to the value $R_{z}=0$ in (2).

A new approach was proposed in the literature to assess the performance of GHG emissions from CHP and CCHP systems [47]. The *TCO_2_ER* indicator was considered to correctly identify the reduction of emissions from combined energy systems compared to conventional benchmarks for separate production (thermal, electric, and cooling). The main characteristics of all of the technological components involved in the study were modeled by means of subsystems from the installation component. The characterization of the emission performance evaluation indicators was made through the terms and emission factors related to the production of different forms of energy. This approach was formulated on the basis of carbon dioxide emissions as the most important amount in the GHG economy. The mathematical model was extended to take into account, in addition to the amount of carbon dioxide, other GHG emissions from cogeneration and trigeneration systems, such as the methane gas contained in gases resulting from the combustion of thermal equipment or leaks of GHG substances from the chillers that use them as refrigerants.

The introduction of some input data, chosen appropriately, to the estimation relation of the *TCO_2_ER* indicator allows carrying out the different case studies and implicitly many analyses from the point of view of the obtained results. As previously shown, this complex indicator is able to assess the effective reduction of all emissions under the general operating conditions of the entire combined energy generation system. Using relevant input data, different simulations can be made, and the results obtained can be used in the case of designing/redesigning different equipment. Additionally, by changing some reference values of the cogeneration/trigeneration system, it is possible to perform different analyses depending on the results obtained. In this context, presented previously, a significant theoretical result refers to the analogy between the *TCO_2_ER* indicator, calculated only based on the level of carbon dioxide emissions (neglecting the contribution of other greenhouse gases) and the other quantitative indicators on the assessment of the level of energy saving in the trigeneration process. Assuming that the same amount of fuel is introduced as entering the combined energy system and the subsystems that make up the production system, and the two indicators have similar numerical results, it can be said that, in this case, according to the developed model, the energy saving and emission reduction of carbon dioxide have the same levels [47,53].

By further defining the *CO_2_EEE* indicator:

(3) $${CO}_{2}EER=\left(\frac{W_{z}}{F_{z}}-\frac{R_{z}}{F_{z}\cdot COP^{SP}}\right)\;\cdot \;\left(1-\frac{{\left(\mu_{{\mathrm{CO}}_{2}}^{Q}\right)}_{SP}}{{\left(\mu_{{CO}_{2}}^{W}\right)}_{SP}}\;\cdot \;\frac{Q_{z}}{F_{z}}\right)\;$$

*CO_2_EEE* can be interpreted as an indicator that refers to the equivalent efficiency of the cogeneration or trigeneration systems. *CO_2_EEE* is an indicator expressed only in terms and factors that characterize the energy performance of the combined system, as well as the emission factors, and *COP^SP^* refers to the separate production of different forms of energy in classical production systems. *COP^SP^* takes the values of 3, 4, and 5 and evaluates the impact of different references for separate cooling generation.

Starting from the theoretical presentation, in the technical literature, case studies were performed using specific analysis techniques and a number of additional indicators were identified. For example, the effectiveness of the proposed techniques and the validation of the evaluation models were illustrated in various case studies on a wide range of cogeneration and trigeneration systems. The current case studies refer to different possible techniques for evaluating reference production systems and are used to calculate the numerical values of the most important parameters specific to different cogeneration and trigeneration technologies [47,53].

**Figure 6:**
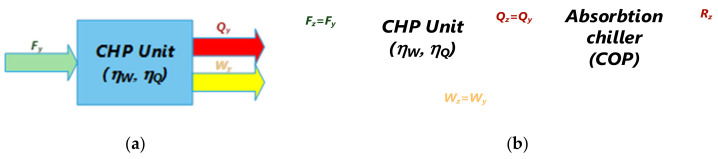
(**a**) Cogeneration system (CHP) scheme; (**b**) trigeneration system (summertime configuration-CCHP) scheme. *F_y_*/*F_z_*—fuel thermal content for CHP/CCHP processes kWht; *Q_y_*/*Q_z_*—heat from CHP/CCHP processes kWht; *R_z_*—cooling from CCHP process (kWht); *W_y_*/*W_z_*—electricity from CHP/CCHP processes (kWhc); *η_W_*/*η_Q_*-electrical/thermal energy efficiency of the CHP/CCHP processes.

Figure 6a shows a system for obtaining different forms of energy through a cogeneration plant modeled as a system (a black box), whose input and output parameters are represented by the relevant energy efficiency (electrical and thermal). Additionally, in Figure 6b, the configuration of a seasonal trigeneration system is modeled (summer time), in which all of the thermal energy obtained from the cogeneration process follows the supply path of an absorption chiller. The components of the system are described by means of subsystems (black boxes) characterized by energy efficiencies (relevant), electrical and thermal energy efficiencies for the cogeneration/trigeneration system, and *COP* for the liquid-absorbed chiller.

If the amount of fuel introduced into the cogeneration system and separate production (*SP*) systems is not the same, the method of analysis presented above is still applied, with only minor adjustments. The carbon dioxide emission factor is closely related to the production of thermal and electrical energy in separate production systems and is expressed as follows:

(4) $${\left(\mu_{{CO}_{2}}^{Q}\right)}_{SP}=\frac{{\left(\mu_{{CO}_{2}}^{F}\right)}_{y}}{\varepsilon_{t}\;\cdot \eta_{t}^{SP}}$$

where:

${\left(\mu_{{CO}_{2}}^{F}\right)}_{y}$-the specific fuel input;

$\varepsilon_{t}$-the correction factor, defined as $\varepsilon_{t}=\frac{{\left(\mu_{{CO}_{2}}^{F}\right)}_{y}}{{\left(\mu_{{CO}_{2}}^{F}\right)}_{SP}}$;

$\eta_{t}^{SP}$-production thermal efficiency.

Thus, for cogeneration, the expressions of *TCO_2_ER* and *CO_2_EEE* become:

(5) $${TCO}_{2}ER=1\;-\frac{\varepsilon_{t}\;\cdot \;\eta_{t\;}^{SP}\;\cdot \frac{{\left(\mu_{{CO}_{2}}^{F}\right)}_{z}}{{\left(\mu_{{CO}_{2}}^{W}\right)}_{SP}}}{\varepsilon_{t\;}\cdot \;\eta_{W}\;\cdot \;\eta_{t}^{SP}+\eta_{Q}\;\cdot \frac{{\left(\mu_{{CO}_{2}}^{F}\right)}_{z}}{{\left(\mu_{{CO}_{2}}^{W}\right)}_{SP}}}$$

(6) $${CO}_{2}EER=\frac{\varepsilon_{t}\;\cdot \;\eta_{W}\;\cdot \;\eta_{t}^{SP}}{\varepsilon_{t}\;\cdot \;\eta_{t}^{SP}+\eta_{Q}}$$

Since the total amount of heat obtained from cogeneration is used only to supply the cooling subsystem $\left(Q_{z}=0\right)$, taking into account those presented above and given that *R_z_ = COP⋅η_Q_⋅F_z_*, the general formulation yields, for trigeneration, the expressions of *TCO_2_ER* and *CO_2_EEE*:

(7) $${TCO}_{2}ER=1-\frac{\frac{{\left(\mu_{{CO}_{2}}^{F}\right)}_{z}}{{\left(\mu_{{CO}_{2}}^{W}\right)}_{SP}}}{\eta_{W}+\eta_{Q}\;\cdot \;\left(\frac{COP}{COP^{SP}}\right)}$$

(8) $${CO}_{2}EER=\eta_{W}+\eta_{Q}\;\cdot \frac{COP}{COP^{SP}}$$

Taking into account the specialized literature, in this section, synthetically new indicators that allow the analysis and evaluation of the characteristics of GHG emissions (and especially carbon dioxide) of different cogeneration and trigeneration systems are presented. The proposed *TCO_2_ER* indicator was used to evaluate the possible decreasing of GHG emissions from the cogeneration and trigeneration systems in terms of separate production of energies (thermal, electric, and cooling) from conventional resources. In particular, the *TCO_2_ER* indicator highlights the potential for different production technologies in terms of emission reductions due to the efficiency resulting from a high level of energy production from cogeneration and trigeneration sources compared to the differences in the reference efficiency of production. For the efficient and correct calculation of the characteristics of cogeneration and trigeneration systems, in the case of the use of general input data sets, the *CO_2_EEE* indicator has been introduced in particular, which represents the equivalent efficiency in terms of reducing carbon dioxide emissions for a given energy level produced by the analyzed systems.

The analysis of processes and indicators is completely general. This analysis also allows for operational assessments, taking into account energy efficiency and quantitative emission characteristics. The results obtained when using different cogeneration and trigeneration systems strongly depend on the technologies used, as well as on the quantities of fuels used for the separate production of thermal energy and especially electricity. The most relevant parameter in performing such analyses is proposed in the *CO_2_EEE* indicator, which highlights the average characteristics of the greenhouse gas emissions produced when obtaining electricity in relation to a reference fuel.

Additionally, as can be seen, the energy efficiency for energy production processes has different values:

a. Classic process efficiency 33% for electricity and 90% for thermal agent;
b. Efficiency of the cogeneration process 85%;
c. Efficiency of the trigeneration process up to 90%.

Depending on the availability and ease of access to fuel sources, it is economically advisable to invest in specific cogeneration and trigeneration technologies because they pay off quickly. Additionally, compared to the classical technologies for obtaining different forms of energy, the two modern technological processes described and studied in this chapter are less polluting, as can be seen from the analysis of the formulas of the proposed indicators for assessing pollution levels.

The method of analysis used by overlapping the two influencing factors (economic efficiency for energy production and reducing greenhouse gas emissions) highlighted the usefulness of obtaining energy through the two modern technologies (cogeneration and trigeneration).

It can also be pointed out that the comparison between the combined generation methods and input references is purely conventional. In particular, assessing the level of reduction of greenhouse gas emissions in relation to the average total emissions generated by electricity production in a given geographical area is a purely theoretical issue. In view of the above, such an approach could be appropriate if there is a national regulatory framework (for providing financial incentives for cogeneration or trigeneration plants with low pollutant emissions), as has already been conducted for high-efficiency cogeneration systems (hydro, photovoltaic, and wind) [54]. An extension of the European Emissions Trading Scheme [46] or the introduction of a similar tax mechanism [47] to promote low-emission technologies could also consider adopting a complex *TCO_2_ER* indicator at the EU level to calculate the relevant emission reduction.

## 4. Conclusions

The transition to a green economy is by far one of the most important tasks for humanity in the following years. Many advances have been made at the political level, and world leaders have the topic on the top of their agenda. Cogeneration and trigeneration are technical solutions recognized in the European Union as being economically efficient in the complex process of energy transition. As a result, cogeneration and trigeneration have caught the attention of policymakers as an alternative to increase the efficiency of older processes used to obtain energy as the transition to the green economy is underway. This paper aimed to analyze from the point of view of the two most important influencing factors, the economic efficiency of processes and the reduction of greenhouse gas emissions by overlapping their effect in case of the use of modern technologies (cogeneration and trigeneration) for the combined production of various forms of energy.

The analysis of the indicators leads us to the following important conclusions:

1. The energy yields specific to these technical solutions for cogeneration and trigeneration are good and very good in relation to obtaining separate forms of energy from burning wood, hydrocarbons, and natural gas, because the same volume of fuel is consumed to produce two or even three forms of energy.
2. The reduction of pollutant emissions can be significant due to the same fact, namely that the same volume of fuel is consumed to produce two or even three forms of energy. Additionally, this reduction depends essentially on the technological level of the cogeneration and trigeneration equipment, being influenced by the specific efficiencies of these equipment types. The authors are aware of the limits of the research carried out given the complexity of the energy transition process, and its multiple economic, social, and technical facets. For this reason, a future direction of research is the analysis of the potential of renewable energy in the transition to the green economy.

## Figures and Tables

**Table 1:** Aggregate indicators of the energy transition.

Indices	Objectives/Methodological Coordinates
International Energy Agency’s Energy Development Index (EDI) Multidimensional Energy Poverty Index (MEPI) Sustainable Energy Development Index (SEDI)	Reflects the relationship between human development and access to energy
The World Bank’s Regulatory Indicators for Sustainable Energy	Classifies countries according to the extent to which their policies and legislative framework influence access to energy, energy efficiency, and renewable energy.
The World Energy Council’s Energy Trilemma Index	Assesses countries’ ability to harmonize energy security imperatives with environmental sustainability and energy equity
The Energy Security Index from Global Energy Institute	Measures energy security risks for energy-intensive countries
The Climate Action Tracker	Assesses the progress of countries in helping to achieve the objectives of the Paris Agreement; provides useful indicators for monitoring energy security at the country level
PBL Netherlands Environmental Assessment Agency’s Climate Pledge tool	
The World Economic Forum’s Energy Transitions Index (ETI)	Aggregates 40 variables covering a wide range of energy transition dimensions

Source: Processed personally based on Harsh Vijay Singh et al., 2019 [43].

**Table 2:** Dimensions and variables of the Energy Transition Index (ETI).

System Performance score (50%)	Economic Development and Growth (33%)
	Environmental Sustainability (33%)
	Energy Access & Security (33%)
Transition Readiness score (50%)	Capital& Investment (17%)
	Regulation & Political Commitment (17%)
	Institutions and Governance (17%)
	Infrastructure & Innovative Business Environment (17%)
	Human Capital (17%)
	Energy System Structure (17%)

Source: World Economic Forum, Fostering Effective Energy Transition, 2020 edition [44].

**Table 3:** ETI ranking 2020.

	Country Name	2020 ETI Score	System Performance	Transition Readiness
**1**	Sweden	74.2%	79%	69%
**2**	Switzerland	73.4%	77%	70%
**3**	Finland	72.4%	71%	74%
**4**	Denmark	72.2%	69%	76%
**5**	Norway	72.2%	81%	63%
**6**	Austria	70.5%	70%	71%
**7**	United Kingdom	69.9%	72%	68%
**8**	France	68.7%	74%	64%
**9**	Netherlands	68.0%	68%	68%
**10**	Iceland	67.3%	74%	61%
**11**	Uruguay	67.0%	75%	59%
**12**	Ireland	66.9%	69%	65%
**13**	Singapore	65.9%	67%	65%
**14**	Luxembourg	65.1%	62%	68%
**15**	Lithuania	65.1%	71%	59%
**16**	Latvia	64.9%	69%	60%
**17**	New Zeeland	64.6%	73%	57%
**18**	Belgium	64.5%	65%	64%
**19**	Portugal	64.2%	69%	59%
**20**	Germany	63.9%	64%	64%
**21**	Estonia	63.3%	64%	63%
**22**	Japan	63.2%	64%	63%
**23**	Slovenia	63.1%	66%	60%
**24**	Spain	62.9%	67%	59%
**25**	Colombia	62.7%	72%	54%
**26**	Italy	62.0%	68%	56%
**27**	Costa Rica	61.9%	72%	52%
**28**	Canada	61.7%	67%	56%
**29**	Chile	61.1%	65%	57%
**30**	Israel	60.8%	66%	56%
**31**	Hungary	60.7%	66%	55%
**32**	United States	60.7%	66%	56%
**33**	Slovak Republic	60.5%	66%	55%
**34**	Malta	60.4%	65%	56%
**35**	Romania	59.9%	68%	52%
**36**	Australia	59.7%	66%	54%
**37**	Croatia	59.7%	66%	54%
**38**	Malaysia	59.4%	64%	55%
**39**	Peru	59.2%	69%	49%
**40**	Panama	58.9%	66%	52%
**41**	Georgia	58.8%	61%	57%
**42**	Czech Republic	58.5%	61%	56%
**43**	Paraguay	58.4%	68%	49%
**44**	Azerbaijan	58.1%	67%	49%
**45**	Ecuador	58.1%	72%	45%
**46**	Cyprus	58.0%	63%	53%
**47**	Brazil	57.9%	69%	46%
**48**	Korea, Rep.	57.7%	59%	57%
**49**	Brunei Darussalam	57.0%	66%	48%
**50**	Mexico	56.5%	64%	49%
**51**	Morocco	56.5%	61%	51%
**52**	Albania	56.5%	63%	50%
**53**	Thailand	56.3%	61%	51%
**54**	Qatar	56.1%	60%	52%
**55**	Sri Lanka	55.8%	65%	46%
**56**	Argentina	55.8%	68%	44%
**57**	Philippines	55.3%	62%	49%
**58**	El Salvador	55.3%	61%	50%
**59**	Greece	55.0%	63%	47%
**60**	Armenia	54.9%	60%	49%
**61**	Bulgaria	54.2%	59%	49%
**62**	Montenegro	54.2%	55%	53%
**63**	United Arab Emirates	54.0%	56%	52%
**64**	Namibia	53.6%	54%	53%
**65**	Vietnam	53.5%	57%	50%
**66**	Ghana	53.2%	59%	47%
**67**	Turkey	53.1%	57%	49%
**68**	Bolivia	53.0%	64%	42%
**69**	Poland	52.9%	57%	48%
**70**	Indonesia	52.4%	61%	44%
**71**	Dominican Republic	52.4%	59%	46%
**72**	Republic of Moldova	52.4%	61%	43%
**73**	Oman	52.1%	54%	50%
**74**	India	51.5%	54%	49%
**75**	Jamaica	51.5%	54%	49%
**76**	Guatemala	51.2%	58%	45%
**77**	Trinidad and Tobago	50.9%	58%	44%
**78**	China	50.9%	50%	52%
**79**	Kenya	50.6%	47%	54%
**80**	Russian Federation	50.5%	63%	38%
**81**	Tajikistan	49.8%	49%	51%
**82**	Jordan	49.8%	46%	53%
**83**	Algeria	49.1%	61%	37%
**84**	Egypt, Arab Rep.	49.1%	52%	46%
**85**	Honduras	49.0%	51%	47%
**86**	Saudi Arabia	48.7%	54%	43%
**87**	Bangladesh	48.4%	54%	43%
**88**	Kazakhstan	48.3%	59%	48%
**89**	Tunisia	48.2%	53%	43%
**90**	Bahrain	48.1%	46%	51%
**91**	Cambodia	47.8%	49%	47%
**92**	Tanzania	47.4%	47%	48%
**93**	Kuwait	46.9%	52%	42%
**94**	Pakistan	46.6%	46%	47%
**95**	Nepal	46.3%	45%	47%
**96**	Nicaragua	46.1%	50%	42%
**97**	Ethiopia	45.9%	47%	45%
**98**	Zambia	45.7%	47%	45%
**99**	Botswana	44.7%	45%	44%
**100**	Serbia	44.3%	50%	39%
**101**	Iran, Islamic Rep.	43.5%	55%	32%
**102**	Ukraine	43.3%	50%	37%
**103**	Bosnia Herzegovina	43.2%	47%	39%
**104**	Senegal	43.1%	39%	47%
**105**	Kyrgyz Republic	42.7%	42%	43%
**106**	South Africa	42.7%	47%	38%
**107**	Zimbabwe	42.6%	41%	45%
**108**	Mongolia	72.1%	45%	39%
**109**	Mozambique	42.0%	47%	37%
**110**	Benin	41.5%	41%	42%
**111**	Venezuela	41.2%	55%	27%
**112**	Cameroon	41.0%	40%	42%
**113**	Nigeria	40.5%	46%	35%
**114**	Lebanon	38.5%	36%	41%
**115**	Haiti	36.0%	35%	37%
	Advanced economies			
	Commonwealth of independent states			
	Emerging and developing Asia			
	Emerging and developing Europe			
	Latin America and the Caribbean			
	Middle East, North Africa and Pakistan			
	Sub-Saharan Africa			

Note 1: The Energy Transition Index benchmarks countries on the performance of their energy system, as well as their readiness for transition to a secure, sustainable, affordable, and reliable energy future. Note 2: ETI 2020 score on a scale from 0% to 100%. Source: World Economic Forum, Fostering Effective Energy Transition, 2020 edition [44].

**Table 4:** Cogeneration vs. trigeneration.

Technology	Cogeneration	Trigeneration
Definition:	Named in the literature as the process of generating electricity and heat simultaneously/combined simultaneously (CHP), cogeneration consumes a single fuel and achieves production of heat and energy integrated in a single process. The end result is materialized by a system of the cogeneration of electricity and the capture of an amount of the residual heat that it transforms into useful energy. As can be deduced, this method is a much more efficient technical solution than the current method of generating energy, in which heat is released into the atmosphere and is simply blown by the wind from the massive chimneys.	The technological process of trigeneration differs significantly from cogeneration. These technological systems use similar cogeneration units, but are additionally equipped with absorption chillers, so there is the option to provide simultaneous cooling with electricity and heating. The trigeneration process is certainly recommended option in cases when cooling is also required.
Work process:	The cogeneration process involves the generation of two types of energy (electricity and heat) from the consumption of a single fuel source, which excludes the use of other additional heating systems. During the cogeneration process, electricity is generated by a gas turbine generator and the residual heat from the turbine exhaust system is captured and introduced to a heat exchanger. With the help of the heat exchanger, a thermal agent (steam or hot water) can be generated.	The units start with a traditional cogeneration system, coupled with the absorption refrigeration system mentioned above. During this process, the hot water coming from the cooling circuit of the heat exchanger of the cogeneration plant will act as the driving energy for the cooling unit. The gas turbine plays the same role as that in the cogeneration process and continues to evacuate thermal energy that can be used as an energy source. Organizations using technological trigeneration systems can achieve a transformation efficiency similar to that of a cogeneration system and sometimes even higher. With the cooling rate introduced, there is a very good chance that expenses will be able to be easily reduced in the summer months with high temperatures.

Source OGA, Optimal Group Australia, 2016, https://www.optimalgroup.com.au/2016/06/01/understanding-cogeneration-and-trigeneration (accessed on 20 October 2021) [48].

**Table 5:** Comparison between cogeneration energy production systems.

Technical Parameters	Boiler/Steam Turbine	Combined Cycle	Combustion Turbine	Fuel Cell	Microturbine
Electrical efficiency %	15–38%	28–42%	22–36%	30–63%	18–27%
Thermal efficiency %	42–65%	42–43%	39–48%	17–25%	47–48%
Overall efficiency %	80%	70–85%	70–75%	55–80%	65–75%
Availability %	~100%	92–97%	90–98%	>95%	90–98%
Investment cost $/Whe	0.45–1.1	1.1–2.2	0.97–1.3	5–6	2.4–3
Operating cost $/Whe	<5	9–2.2	4–1.1	3.2–3.8	1.2–2.5

Source: [49,50,51].

**Table 6:** Advantages and disadvantages of cogeneration energy production systems.

	Boiler/Steam Turbine	Combined Cycle	Combustion Turbine	Fuel Cell	Microturbine
Advantage	✔ Good efficiency ✔ Large range of fuels Good reliability	✔ Quick start ✔ Investment accessible for small and medium powers ✔ Good climbing with load Does not require auxiliary constructions	✔ Good reliability ✔ Low emissions High temperature heat	✔ Noise and low emissions ✔ Modular design Constant efficiency for the variation of load	✔ Compact design ✔ Few moving parts ✔ Low emissions No cooling required
Disadvantages	✔ Slow start Low electrical/thermal ratio	Low thermal temperature	✔ Requires high pressure for the natural gas or local compressor Low efficiency for the variation of load	✔ Very high investment cost ✔ Relatively short lifecycle Requires special fuel processing	✔ Long-term return on investment Low mechanical efficiency

Source: [49,50,51].

## Data Availability

The data used in this study are public information.
